# Impact of Electronic Prescribing on Parkinson’s Disease Medication Management: A Retrospective Before-and-After Audit at a UK Teaching Hospital

**DOI:** 10.7759/cureus.97619

**Published:** 2025-11-23

**Authors:** Yad Z Omer, Mayurika Chakraborty, Katie Phillips, Gobeka Ponniah, Lucy Strens

**Affiliations:** 1 Internal Medicine, George Eliot Hospital NHS Trust, Nuneaton, GBR; 2 Geriatrics, University Hospital Coventry and Warwickshire, Coventry, GBR; 3 Neurology, University Hospital Coventry and Warwickshire, Coventry, GBR

**Keywords:** electronic health record system, hospital neurology, medication management, parkinsons disease, parkinson’s disease (pd)

## Abstract

Background: Parkinson’s disease (PD) medications are classified as time-critical, with delays or omissions potentially leading to increased morbidity, prolonged admissions, and worsened clinical outcomes. The introduction of electronic prescribing (EPR) has shown potential to reduce prescribing errors, but its impact on PD-specific medication safety remains unclear. This audit aimed to evaluate the accuracy and timeliness of PD medication prescribing and administration before and after the implementation of EPR at a UK teaching hospital.

Methods: A retrospective clinical audit was conducted at University Hospitals Coventry and Warwickshire NHS Trust, Coventry, England. Records from adult inpatient admissions with a confirmed diagnosis of PD were reviewed over two six-month periods: July-December 2023 (pre-EPR) and July-December 2024 (post-EPR). A total of 100 randomly selected admissions (50 from each period) were analysed. Data were collected on prescribing accuracy (dose and formulation), administration timing, missed/delayed doses, and use of dopamine-blocking medications. The audit was assessed against the National Institute for Health and Care Excellence (NICE) QS164, NG71, and QS120 standards, which expect 100% accurate prescribing and administration of time-critical PD medicines within ±30 minutes. Data were analysed using descriptive statistics in Microsoft Excel (Microsoft Corporation, Redmond, WA).

Results: Prescription of the correct PD dose decreased from 48/50 (96%) in 2023 to 45/50 (90%) in 2024, and correct formulation from 48/50 (96%) to 44/50 (88%). Correct administration times fell from 39/50 (78%) to 33/50 (66%). In both years, 36/50 (72%) of patients received their first dose on time. Full adherence to all prescribed doses declined from 28/50 (56%) to 24/50 (48%) post-EPR. Dopamine-blocking medication use increased from 1/50 (2%) to 2/50 (4%). Discharge to care homes increased in 2024 (17/50 (34%) vs. 10/50 (20%)), while in-hospital deaths rose slightly (4/50 (8%) vs. 3/50 (6%)).

Conclusion: Following the implementation of EPR, reductions were observed across several PD medication safety metrics; however, these findings represent observational associations and do not imply direct causation. The results highlight the importance of targeted staff training, early recognition of PD during clerking, and the use of clinical decision-support tools to optimise the safe and effective use of EPR systems in this patient group.

## Introduction

With an increasingly ageing population, the prevalence of Parkinson’s disease (PD) continues to rise [1]. As such, it is more crucial than ever to ensure the effective treatment and management of this progressive neurological condition. Parkinson’s medications are classified as time-critical, as delays in administration or omission of doses can significantly impact patient morbidity and mortality [2]. These delays are associated with prolonged hospital admissions and a subsequent increase in healthcare costs [3].

The literature consistently highlights that prescribing and administration errors frequently occur during unplanned hospital admissions involving patients with PD [4]. The causes of these errors appear to be multifactorial and include delayed medication reconciliation, unavailability of medication on the ward, staffing shortages, increased workload pressures, and limited staff awareness or training on the time-critical nature of Parkinson’s therapy [5,6].

The implementation of electronic prescribing systems has demonstrated considerable improvements in reducing both prescribing and administration errors [7]. However, the success of these systems appears to depend on multiple factors, some of which were identified in this audit, including timely recognition of PD on admission, early prescribing during the initial clerking process, staff training and competence in using the electronic system, and the accurate selection of medication formulations and administration times. In addition, PD medications are available in multiple specialised formulations (e.g., dispersible, controlled-release), and regimens often involve highly individualised dosing intervals. Prescribers may not always be familiar with how to locate or select the correct formulation or accurately specify time-critical administration schedules within the electronic system, increasing the potential for prescribing inaccuracies.

## Materials and methods

This retrospective clinical audit evaluated the management of PD medications - specifically dopaminergic therapies, such as levodopa, dopamine agonists, and monoamine oxidase B (MAO-B) inhibitors - during acute hospital admissions, comparing performance before and after the implementation of EPR.

The primary objective was to assess adherence to the National Institute for Health and Care Excellence (NICE) quality standards across four predefined audit criteria: (1) correct prescription of pre-admission PD medication dose; (2) correct prescription of formulation; (3) timely administration of prescribed doses, including first inpatient dose; and (4) avoidance of dopamine-blocking medications. Additional clinical and demographic data were collected to support contextual interpretation.

Data were collected over two six-month periods: July-December 2023 (pre-EPR) and July-December 2024 (post-EPR) from University Hospitals Coventry and Warwickshire NHS Trust (UHCW), Coventry, England. Inclusion criteria were all adult admissions during the audit periods with a confirmed diagnosis of PD, identified using diagnostic code “G20X” and EPR alert data. Exclusion criteria were duplicate records, admissions without an overnight stay, and diagnoses other than idiopathic PD (e.g., atypical Parkinsonism, vascular Parkinsonism, Alzheimer’s disease).

Following exclusion, 255 eligible admissions remained. From these, 50 admissions from each period were selected for detailed review (total n=100). Although the intention was to obtain a representative sample, a formal randomisation procedure was not applied; therefore, the sample should be regarded as a pragmatic convenience sample.

Data were extracted by a multidisciplinary team comprising three resident doctors, two advanced clinical practitioners (ACPs), and one trainee ACP, using a standardised audit tool developed in accordance with NICE guidance (QS164 [8], NG71 [9], QS120 [10]). Timely administration was defined as delivery within ±30 minutes of the prescribed dosing time in accordance with NICE standards. Data analysis was performed using descriptive statistics in Microsoft Excel (Microsoft Corporation, Redmond, WA). As this was a registered clinical audit, ethical approval was not required; however, institutional governance approval was obtained, and patient confidentiality was maintained throughout.

## Results

A total of 255 admissions to UHCW met the inclusion criteria between July and December 2023 and July and December 2024. From these, 50 admissions were randomly selected from each period for audit and analysis.

**Figure 1:**
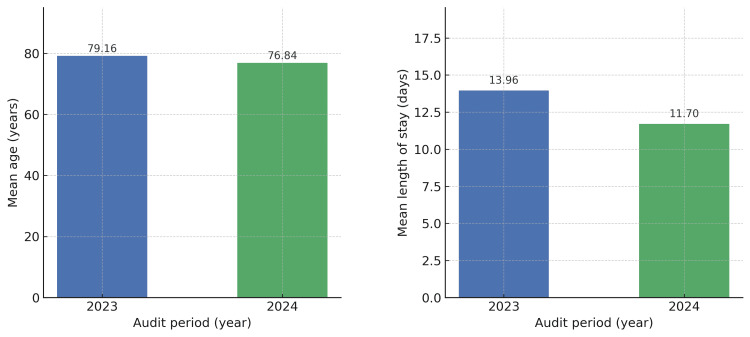
Mean Age and Length of Hospital Stay

**Figure 2:**
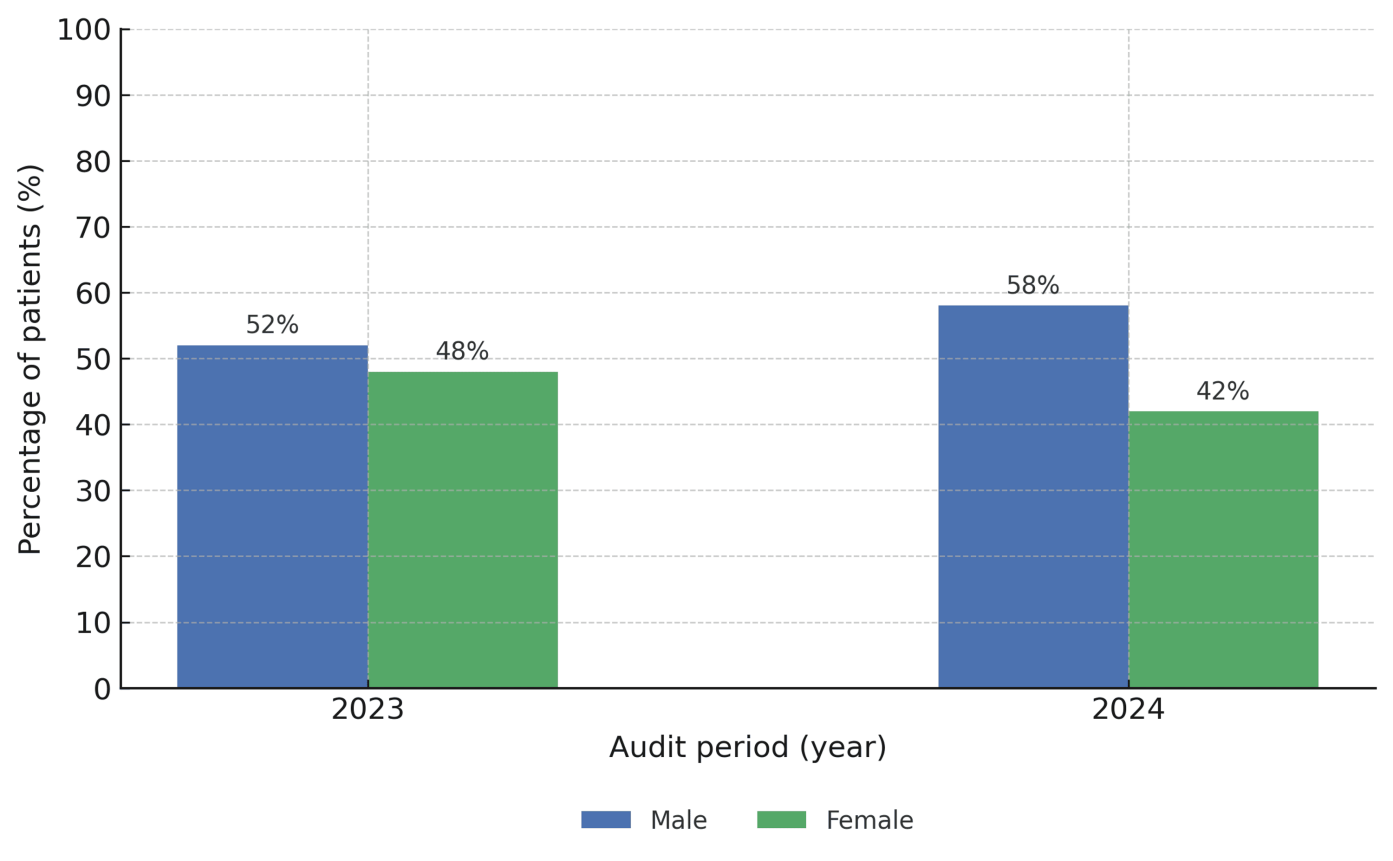
Sex Distribution

The mean age of patients in the 2023 cohort was 79.16 years, compared to 76.84 years in 2024. The mean length of hospital stay was 13.96 days in 2023 and 11.70 days in 2024. In terms of sex distribution, 26/50 (52%) of the 2023 cohort were male, and 24/50 (48%) female, while in 2024, 29/50 (58%) were male and 21/50 (42%) female (Figures 1-2).

**Figure 3:**
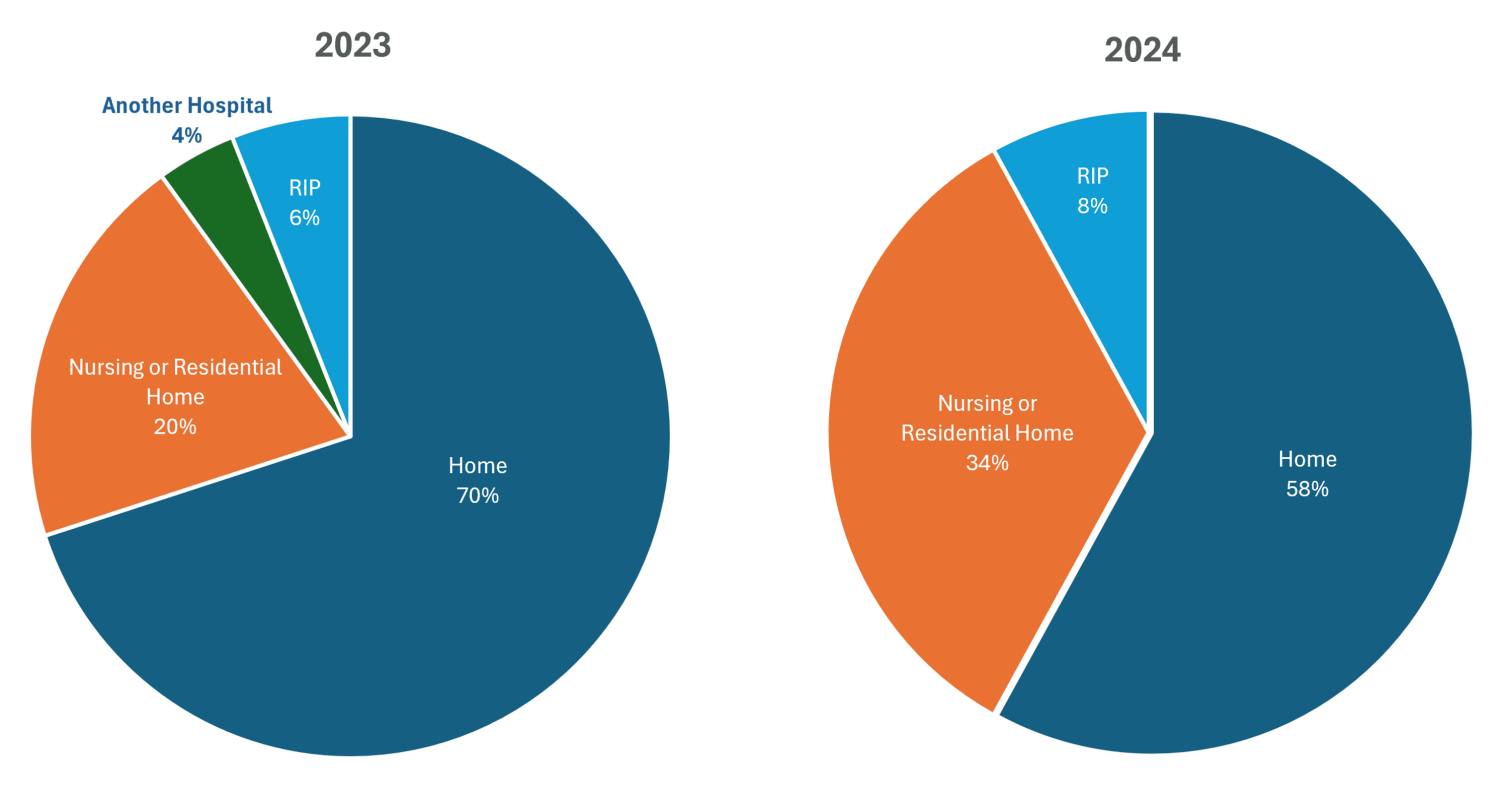
Discharge Destinations

Discharge destinations differed between the two years. In 2023, 35/50 (70%) of patients were discharged to their own homes, 10/50 (20%) to a nursing or residential home, 2/50 (4%) were transferred to another hospital, and 3/50 (6%) died during their hospital admission. In 2024, 29/50 (58%) were discharged home, 17/50 (34%) to a care facility, and 4/50 (8%) died during admission (Figure 3). The most commonly recorded diagnosis on discharge summaries in both years was falls, accounting for 13/50 (26%) of audited admissions.

PD medication management

With regard to PD medication prescribing, 48/50 (96%) of patients in 2023 were prescribed the correct dose, compared to 45/50 (90%) in 2024. The correct formulation was prescribed in 48/50 (96%) of 2023 admissions and in 44/50 (88%) of 2024 admissions. Correct administration times were prescribed in 39/50 (78%) of 2023 admissions and in 33/50 (66%) of 2024 admissions. In both years, 36/50 (72%) of patients received their first dose of PD medication on time following admission.

**Figure 4:**
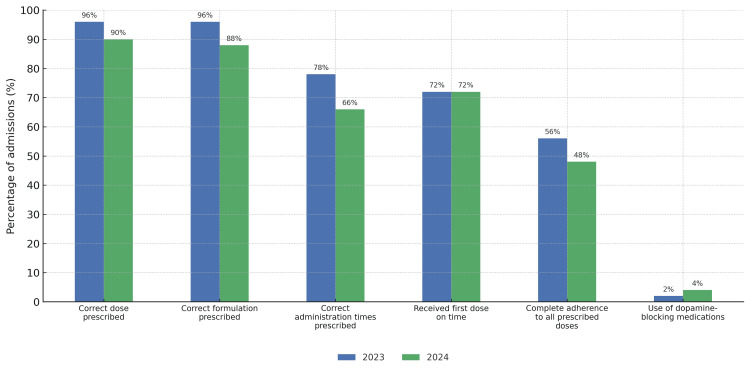
Parkinson's Disease Medication Management Across 2023 and 2024

Complete adherence to administering every prescribed dose of PD medication during the inpatient stay was achieved in 28/50 (56%) of 2023 admissions and 24/50 (48%) of 2024 admissions. Dopamine-blocking medications were prescribed in 1/50 (2%) of admissions in 2023, and this increased to 2/50 (4%) in 2024. In 2023, the only agent prescribed was levomepromazine, while in 2024, haloperidol and levomepromazine were prescribed (Figure 4).

## Discussion

This audit compared the management of PD medications in acute medical admissions at UHCW before and after the introduction of an EPR system in 2024. A total of 100 admissions (50 from each year) were audited against key quality indicators for PD medication management, including prescription accuracy, administration timing, and use of dopamine-blocking medications contraindicated in PD.

The demographic characteristics of the two cohorts were broadly comparable. The mean age of patients in 2023 was slightly higher at 79.16 years compared to 76.84 years in 2024, and the average length of stay decreased from 13.96 days to 11.70 days following the introduction of EPR. Gender distribution was similar, though 2024 had a higher proportion of male admissions (29/50 (58%) vs. 26/50 (52%)).

Contrary to expectations, the transition to EPR was associated with a decline in the accuracy of PD medication management. The percentage of patients with their correct Parkinson’s medication doses prescribed fell from 48/50 (96%) in 2023 to 45/50 (90%) in 2024. Similarly, the proportion prescribed the correct formulation prescribed decreased from 48/50 (96%) to 44/50 (88%). Correctly prescribed administration times also declined from 39/50 (78%) to 33/50 (66%). These findings highlight that the introduction of an electronic system did not automatically improve prescribing practices and, in some cases, may have introduced new sources of error or inefficiency. Upon investigation, we identified that, during the early days of the electronic system, many users struggled to prescribe the right formulations and to change the administration times on EPR. Since the audit period, there has been ongoing training provided to optimize electronic prescriptions.

Potential confounding factors include the transition phase following EPR implementation, varying staffing levels, pharmacy workflow delays, and medication supply issues. Additionally, prescribing errors reflect input stage mistakes by prescribers, whereas administration errors reflect operational and nursing workflow challenges; these pathways may require targeted interventions independently.

One area where performance remained static was the administration of the first inpatient PD medication dose, with 36/50 (72%) of patients receiving this dose on time in both 2023 and 2024. However, the proportion of patients who received all prescribed doses during their admission declined from 28/50 (56%) to 24/50 (48%) following EPR implementation. This trend raises concerns about inpatient medication continuity, even when prescriptions are technically accurate.

The use of dopamine-blocking medications, such as haloperidol and levomepromazine, increased from 1/50 (2%) in 2023 to 2/50 (4%) in 2024. Given the well-documented risks of dopamine antagonists in PD, these figures are a persistent area of concern and warrant continued education and system alerts.

Discharge destinations showed a shift towards more patients being discharged to care homes in 2024 (17/50 (34%) vs. 10/50 (20%)), with fewer patients returning home (29/50 (58%) vs. 35/50 (70%)). In-hospital mortality also increased from 3/50 (6%) to 4/50 (8%). While these changes may reflect multiple system-level or clinical variables, they raise the possibility that medication management - including delays or inaccuracies in PD medication delivery - may influence functional outcomes and discharge planning.

Not unexpectedly, the most common discharge diagnosis in both years was falls, accounting for 13/50 (26%) in each year. This aligns with existing literature highlighting the strong association between PD, impaired mobility, and fall-related hospitalisations [11].

Overall, these results suggest that, while EPR offers potential advantages in standardization and legibility, its implementation alone does not guarantee improvement in disease-specific prescribing quality. The decline in several key metrics post-EPR highlights the need for targeted clinical decision support tools, robust staff training, and specialty involvement - particularly for complex regimens like those required in PD.

Recommendations

The findings of this audit highlight opportunities to strengthen processes related to the management of PD medications following the implementation of EPR. We recommend enhanced training for medical and nursing staff on the correct prescribing and administration of time-critical PD medications within EPR systems, with particular focus on dose accuracy, formulation selection, and schedule customisation. Incorporating EPR-specific training on time-critical medication prescribing into induction programmes for new prescribers may further support safe practice.

The integration of clinical decision-support features, such as prescribing prompts at admission and automated alerts for incorrect formulations or delayed or missed doses, may help reduce the risk of errors. Collaboration with PD specialist teams during inpatient admissions, particularly early in the admission period, may also support timely optimisation of medication regimens.

Periodic re-audits are recommended to assess improvement over baseline performance, particularly following system updates or education initiatives, with the long-term aim of achieving full compliance with NICE standards. Finally, educational and awareness initiatives should aim to reduce the use of dopamine-blocking medications in patients with PD, supported by appropriate EPR warning alerts.

Limitations

This audit was limited by its small sample size and single-centre design, which may affect generalisability. Data were collected retrospectively and relied on the accuracy of electronic documentation. We did not adjust for potential confounders such as disease severity or staffing levels, and no formal power calculation was performed, as this was a service evaluation.

Disease severity (e.g., Hoehn & Yahr stage or cognitive status) was not captured and may represent an important confounder influencing medication complexity and outcomes.

The patient sample was selected using a pragmatic convenience sampling approach rather than formal randomisation, which may limit generalisability beyond the study population.

## Conclusions

This audit compared the management of PD medications before and after the implementation of a system in an acute hospital setting. While reductions were observed across several key adherence measures following EPR implementation - including correct dosing, formulation selection, administration timing, and the avoidance of dopamine-blocking medications - these findings should be interpreted as observational associations rather than evidence of direct causation. Multiple contextual and operational factors, including workflow changes, prescriber familiarity, and system learning curves, may have contributed to the observed variation.

Overall, the findings emphasise that EPR systems should be viewed as tools that require appropriate configuration, training, and clinical governance to realise their full safety-enhancing potential - particularly for patients prescribed complex and time-critical regimens such as those required in PD. Continued system refinement, targeted education, and specialist collaboration are likely to support improvement in future practice.

## References

[1] Ben-Shlomo Y, Darweesh S, Llibre-Guerra J, Marras C, San Luciano M, Tanner C (2024). The epidemiology of Parkinson's disease. Lancet.

[2] Richard G, Redmond A, Penugonda M, Bradley D (2022). Parkinson’s disease medication prescribing and administration during unplanned hospital admissions. Mov Disord Clin Pract.

[3] Martinez-Ramirez D, Giugni JC, Little CS (2015). Missing dosages and neuroleptic usage may prolong length of stay in hospitalized Parkinson's disease patients. PLoS One.

[4] Magdalinou KN, Martin A, Kessel B (2007). Prescribing medications in Parkinson’s disease (PD) patients during acute admissions to a district general hospital. Parkinsonism Relat Disord.

[5] Saleem A, Ungcharoen N, Bell F, Storton J, Bibi H (2023). Improving Parkinson’s medicine administration in hospitals: the impact of an out-of-hours drug box. Cureus.

[6] Derry CP, Shah KJ, Caie L, Counsell CE (2010). Medication management in people with Parkinson's disease during surgical admissions. Postgrad Med J.

[7] Westbrook JI, Sunderland NS, Woods A, Raban MZ, Gates P, Li L (2020). Changes in medication administration error rates associated with the introduction of electronic medication systems in hospitals: a multisite controlled before and after study. BMJ Health Care Inform.

[8] (2025). Parkinson’s disease: quality standard (QS164). Quality Standard.

[9] (2025). Parkinson’s disease in adults: NICE guideline. https://www.nice.org.uk/guidance/ng71.

[10] (2025). Medicines optimisation: quality standard (QS120). Quality Standard.

[11] Paul SS, Harvey L, Canning CG, Boufous S, Lord SR, Close JC, Sherrington C (2017). Fall-related hospitalization in people with Parkinson's disease. Eur J Neurol.

